# Immune Function Genes, Genetics, and the Neurobiology of Addiction

**DOI:** 10.35946/arcr.v34.3.11

**Published:** 2012

**Authors:** Fulton T. Crews

**Affiliations:** **Fulton T. Crews, Ph.D.,***is a John Andrews Distinguished Professor, professor of pharmacology and psychiatry, and director of the Bowles Center for Alcohol Studies, School of Medicine, University of North Carolina at Chapel Hill, Chapel Hill, North Carolina.*

**Keywords:** Other drug dependence, alcoholism, addiction, causes of alcohol and other drug use, genetic factors, environmental factors, neurobiology, neuroimmune system, immune system, innate immune system, innate immune genes, immune function genes, nuclear factor-κB, stress, decision making, depression

## Abstract

The neuroimmune system (i.e., the immune system and those components of the nervous system that help regulate immune responses), and in particular the innate immune system, play a role in the development of addictions, including alcoholism, particularly in the context of stressful situations. Certain cells of the neuroimmune system are activated both by stress and by environmental factors such as alcohol, resulting in the induction of genes involved in innate immunity. One of the molecules mediating this gene induction is a regulatory protein called nuclear factor-κB, which activates many innate immune genes. Innate immune gene induction in certain brain regions (e.g., the frontal cortex), in turn, can disrupt decision making, which is a characteristic of addiction to alcohol and other drugs. Likewise, altered neuroimmune signaling processes are linked to alcohol-induced negative affect and depression-like behaviors and also regulate alcohol-drinking behavior. Moreover, the expression of several genes and proteins involved in innate immunity is enhanced in addicted people. Finally, specific variants of multiple innate immune genes are associated with the genetic risk for alcoholism in humans, further strengthening the connection between increased brain innate immune gene expression and alcohol addiction.

The nervous system and the immune system interact closely to regulate the body’s immune responses, including inflammatory responses. Accordingly, the term “neuroimmune system” refers to the immune system and those components of the nervous system that help regulate immune responses and also encompasses the hormones and other signaling molecules that convey signals between the immune and nervous systems. Part of the neuroimmune system is the innate immune system—a network of cells and the signaling molecules they release that are present from birth and form the first line of the body’s defense system, including such responses as inflammatory reactions. This article summarizes the role that the neuroimmune system and genes encoding components of the innate immune system play in the development of addiction, including alcoholism.

## Neuroimmune Signaling, Drug Abuse, and Stress

**Figure 1:**
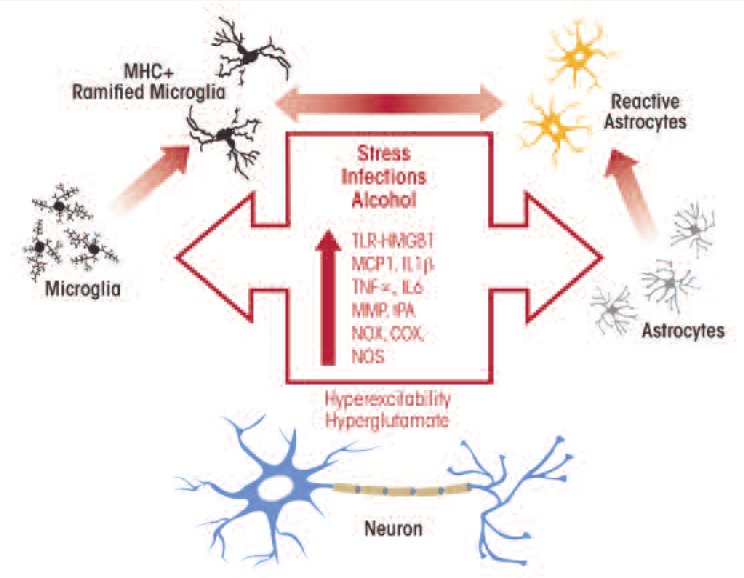
Activation of microglia and astrocytes by alcohol in the brain. Microglia and astrocytes undergo multiple stages of activation that include characteristic changes in morphology. Resting microglia become ramified microglia with that express molecules called major histocompatibility complex (MHC) on their surface. Similarly, astrocytes begin to show markers of reactive astrocytes. Alcohol-induced glial activation is associated with increased expression of innate immune genes, including increased expression of the chemokine monocyte chemoattractant protein-1 (MCP1); the cytokines tumor necrosis factor-α (TNFα), interleukin-1 β (IL-1β), and interleukin-6 (IL-6); the proteases matrix metalloproteinase (MMP) and tissue plasminogen activator (TPA); and the oxidases nicotinamide adenine dinucleotide phosphate oxidase (NOX), cyclooxygenase (COX), and nitric oxide synthetase (NOS). The alcohol-induced activation of glial innate immune genes increases neuronal hyperexcitability (Crews et al. 2011).

Neuroimmune signaling influences the responses and functions of a variety of body systems, including the digestive (i.e., enteric) system, sensory pathways, and the hormonal axis known as the hypothalamic–pituitary–adrenal (HPA) axis, which is involved in the body’s stress response and also plays a role in addiction to alcohol and other drugs (AODs).^1^ Immune cells called monocytes and monocyte-like cells in the brain (e.g., microglia) are sensitive key cells involved in neuroimmune signaling. When the immune system is stimulated or tissue damage occurs, these cells go through multiple stages of activation, which at the molecular level are reflected by the activation of a cascade of innate immune genes (Graeber 2010). These responses of the monocytes and microglia involve the production and secretion of signaling molecules, including inflammation-promoting (i.e., proinflammatory) cytokines and chemokines, such as monocyte chemotactic protein (MCP)-1, tumor necrosis factor α (TNFα), and interleukin 1β (IL1β). In the brain, microglial activation contributes to the activation of another type of cell called astroglia, or astrocytes, which, like microglia, show multiple stages of neuroimmune activation. In the microglia, the different stages of activation are accompanied by morphological changes. Thus, these cells change from their resting state with multiple branches (i.e., the ramified form) to a less branched, bushy morphology after mild activation and a rounded morphology after strong activation (i.e., when major brain cell death occurs). Chronic alcohol treatment induces mild, bushy microglial activation as well as mild astrocyte activation (see figure 1).

Activated glia show increased production of a wide range of proteins. For example, they produce and secrete increased amounts of proteases as well as of proteins found in the space between cells (i.e., extracellular matrix proteins). In addition, they generate increased amounts of proteins called toll-like receptors (TLRs) that play a role in alcohol-induced depressed mood and negative emotions (see below) and show enhanced activity of enzymes known as oxidases that promote inflammatory reactions (e.g., nicotinamide adenine dinucleotide phosphate [NADPH] oxidases [NOX], cyclooxygenase [COX], and inducible nitric oxide synthases [iNOS]).

**Figure 2:**
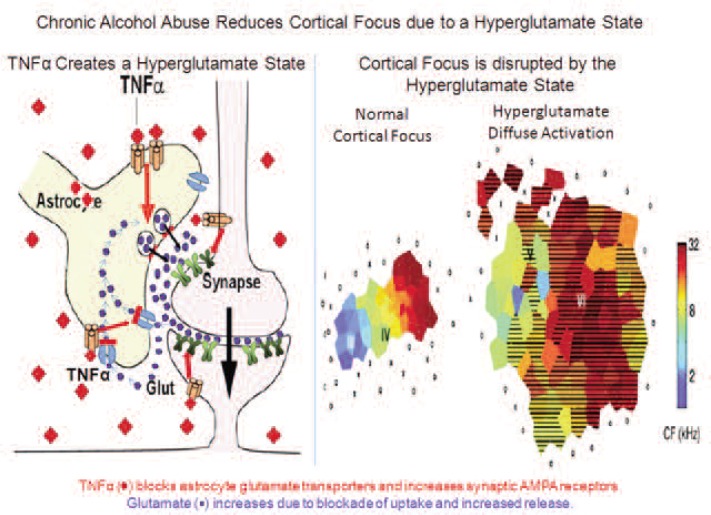
Mechanisms of alcohol-induced excessive glutamate activity in the cortex and loss of cortical focus. Ethanol-induced activation of microglia and astrocytes increases the levels of proinflammatory cytokines, including tumor necrosis factor-alpha (TNFα). (Left panel) TNFα creates a state characterized by excess activity of the neurotransmitter glutamate (i.e., a hyperglutamate state). Thus, TNFα reduces the levels of the primary glutamate transporters, GLT-1, in the astrocytes, in the cerebral cortex, and inhibits glutamate transport, possibly through induction of TNFα and other proinflammatory genes. As a result, glutamate levels outside the neurons, and particularly at the synapse, increase, resulting in a hyperglutamate state. In addition, TNFα increases the levels of certain molecules that interact with glutamate (i.e., AMPA receptors). All these processes causes excessive neuronal excitability. (Right panel) Hyperexcitability disrupts cortical focus. The left image shows the response of a normal adult auditory cortex to a series of tones with a frequence of 2–32 kHz colorized as blue to red. The response to a specific tone involves activation of a specific focal cortical region, which likely relates to the ability to distinguish specific tones of sounds. The right image shows the disrupted hyperglutamate-state–like response to sound that involves the entire auditory cortex without specific tonal areas of focus. The hyperglutatamate state increases cortical excitability, which in turn decreases function because it results in loss of focal activation and likely loss of tonal discrimination. In alcoholism, the hyperglutamate state most strongly affects the frontal cortex, which may disrupt decision making as well as attention and behavioral control mechanisms. SOURCE: Image in right panel adapted from Chang and Merzenich (2003).

Microglia easily can become activated, and the initial stages of activation are characterized by the secretion of signaling molecules, slight morphological changes, and increased production of molecules involved in immune responses (i.e., major histocompatibility complex [MHC]) as well as of TLRs (Graeber 2010). Activation of microglia and astrocytes also increases proinflammatory agents, including TNFα, that alter the transmission of nerve signals (i.e., neurotransmission), including signal transmission mediated by the excitatory neurotransmitter glutamate. Likewise, studies have suggested that alcoholism is related to excessive glutamate levels (i.e., a hyperglutamate state). In the outer layer of the brain (i.e., the cerebral cortex), chronic alcohol-induced neuroimmune activation leads to a hyperglutamate state that reduces cortical function (figure 2). One mechanism contributing to this hyperglutamate state involves TNFα, which acts to reduce the activity of glutamate transporters^2^ in the astrocytes (Zou and Crews 2005). Similarly, beverage alcohol (i.e., ethanol) has been shown to inhibit glutamate transport (Zou and Crews 2006). This blockade of glutamate transporters increases glutamate levels outside the cells and particularly in the space between two neurons where nerve signals are transmitted (i.e., the synapse), resulting in excessive neuronal activity (i.e., hyperexcitability). TNFα also stimulates the production of certain proteins found on signal-receiving neurons that interact with glutamate (i.e., the AMPA glutamate receptors) (Beattie et al. 2010). Increases in synaptic glutamate receptors and glutamate concentrations cause hyperexcitability that disrupts the normal concentration of the brain’s response to a specific area of the cortex (i.e., cortical focus), thereby reducing cortical function. Through these mechanisms, monocytes, microglia, and astrocytes progressively become activated by stress and environmental factors, including ethanol, resulting in the induction of genes that encode proteins involved in the innate immune response.

## Stress and Drug Abuse Increase Transcription of Innate Immune Genes

**Figure 3:**
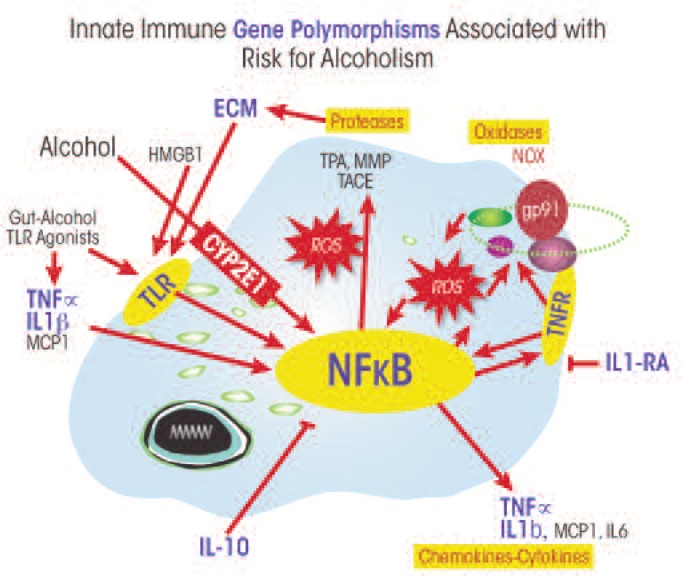
Innate immune gene polymorphisms associated with risk for alcoholism. The schematic shows a representative astrocyte or microglial cell. Genes associated with genetic risk for alcoholism are in light blue. Nuclear factor κ-lightchain–enhancer of activated B cells (NF-κB) is a key transcription factor involved in induction of innate immune genes that is sensitive to reactive oxygen species (ROS). These ROS are generated by the enzyme CYP2E1 during alcohol metabolism, and certain DNA sequences (i.e., polymorphisms) in the CYP2E1 gene are associated with alcoholism. CYP2E1 is highly expressed in monocyte-like cells, which are activated when CYP2E1 metabolizes alcohol. The ROS formed during this process activate proinflammatory NF-κB responses. Chronic ethanol treatment increases CYP2E1 expression in the brain, particularly in astrocytes.The resulting elevated ROS levels activate NF-κB–mediated transcription of innate immune genes, and this response may be amplified in the presence of certain NF-κB polymorphisms (i.e., NF-κB1). Certain variants of other genes also are associated with alcoholism, including polymorphisms of T NFα, interleukin-10 (IL-10), interleukin-1 receptor antagonist (IL-1RA), and other components of the IL-1 gene complex, as well as of certain proteins in the space surrounding the cells (i.e., extracellular matrix proteins [ECM]).

Stress and AODs, as well as sensory and hormonal signals, activate a regulatory protein (i.e., transcription factor^3^) called nuclear factor κ–light-chain enhancer of activated B cells (NF-κB) that is produced in large amounts (i.e., is highly expressed) in monocytes and microglia. Although NF-κB is found in most cells, it is the key transcription factor involved in the induction of innate immune genes in microglia and other monocyte-like cells. A wide range of stimuli, such as stress, cytokines, oxidative free radicals, ultraviolet irradiation, bacterial or viral molecules, and many other signaling molecules, increase binding of NF-κB to specific sequences of the DNA. This binding increases the transcription of many genes, particularly those encoding signaling molecules (e.g., chemokines and cytokines) and enzymes (e.g., oxidases and proteases) (figure 3). Studies found that ethanol can increase the binding of NF-κB to its corresponding DNA sequences both in the brains of living organisms (Crews et al. 2006) and in cultured brain slices obtained from a brain area called the hippocampal–entorhinal cortex (HEC) (Zou and Crews 2006). These and other studies also have indicated that ethanol increases transcription of NF-κB target genes, including the genes encoding the following:

- MCP-1;
- Certain proinflammatory cytokines, such as TNFα, IL-1β, and IL-6;
- Certain proinflammatory oxidases, such as iNOS (Zou and Crews 2010), COX-2 (Knapp and Crews 1999), and NOX (Qin et al. 2008); and
- Certain proteases, such as TNF–converting enzyme (TACE) and tissue plasminogen activator (Zou and Crews 2010).

Not only ethanol but also chronic stress increases brain NF-κB activation (Koo et al. 2010; Madrigal et al. 2002), as well as the levels of cytokines, prostaglandin,^4^ and COX-2 (Madrigal et al. 2003), all of which have proinflammatory effects. Although acute stress–induced responses, such as elevated glucocorticoid levels, are anti-inflammatory by blocking NF-κB production, chronic elevation of glucocorticoid levels during cycles of stress and/or AOD abuse reverses these anti-inflammatory effects and indeed results in proinflammatory NF-κB activation in the frontal cortex (Munhoz et al. 2010). Thus, activation of NF-κB is a common molecular mechanism through which stress and AODs can induce innate immune genes.

## Addiction and Neuroimmune Signaling

Alcoholism is a progressive disease related to repeated episodes of alcohol abuse that reduce the brain’s behavioral control and decision-making ability; at the same time, increasing habitual urges combined with increasing bad feelings (i.e., negative affect) promote continued drinking. Frontal cortical brain regions that designate attention and motivation, using information to predict the result of actions (Schoenbaum and Shaham 2008), play a role in addiction development. Frontal cortical dysfunction often is investigated using reversal-learning tasks. In reversal learning, the subject first learns to make one choice (e.g., responding to the black objects in a series of black and white objects) and then has to learn to reverse this choice (e.g., to respond to the white objects). Thus, the initially expected responses suddenly are considered wrong, requiring the subject to exhibit flexible behavior in response to outcomes that do not match those predicted by preceding cues (Stalnaker et al. 2009).

In behavioral studies, poor performance on such tasks is supposed to reflect the inability of drug-addicted individuals to learn new healthy behaviors and avoid the negative consequences of their drug consumption. Such learning and/or changes in behavior require signals from the frontal cortex to indicate the value of decisions. Studies found that binge drinking induces persistent deficits in reversal learning in rats (Obernier et al. 2002; Pascual et al. 2007) and in adult mice following a model of adolescent binge drinking (Coleman 2010). Other investigators similarly have demonstrated that cocaine use results in abnormally slow reversal learning, even though initial learning is normal (Calu et al. 2007; Schoenbaum et al. 2004). Specifically, human cocaine and alcohol addicts exhibit dysfunctional decision making in reversal-learning tasks that probe cognitive flexibility (Bechara et al. 2002). Lesions in the frontal cortex cause reversal-learning deficits comparable to those induced by chronic drug abuse (Schoenbaum et al. 2006). The persistence of addiction matches the persistent increases in innate immune gene activation (Qin et al. 2007, 2008) and loss of behavioral flexibility. Thus, it is thought that innate immune gene induction in the frontal cortex disrupts decision making consistent with addiction (Crews et al. 2011).

Addiction to alcohol, opiates, and stimulant drugs involves both changes in attention–decision making and increased temporal lobe anxiety–negative affect urgency. Addiction-induced negative affect and depression-like behaviors also are linked to neuroimmune signaling because neuroimmune signals can alter moods. For example, a compound called lipopolysaccharide (LPS) that can induce brain innate immune genes causes depression-like behavior that mimics components of addiction-like negative affect. LPS naturally binds with one of the TLRs (i.e., TLR4) and this interaction results in NF-κB activation, ultimately leading to the induction of innate immune genes. In humans, LPS infusions reduce reward responses and increase depressed mood (Eisenberger et al. 2010). Likewise, when patients with cancer or viral infections are treated with agents such as interferon and IL that influence innate immune genes, they may experience severe depression as a major adverse effect (Kelley and Dantzer 2011). Innate immune activators such as LPS, chemokines, and cytokines can mimic the amplification of depressed mood that occurs during repeated cycles of drug abuse or stress (Breese et al. 2008). All of these observations further support the link between neuroimmune signaling and mood as well as the role of neuroimmune signaling as a key component of addiction neurobiology. Of interest, chronic alcohol leads to withdrawal anxiety in normal micetent with the hypothesis that innate immune activation drives negative affect and associated anxiety responses. Thus, the anxiety–depression negative affect that contributes to addiction occurs with increased brain neuroimmune signaling.

Neuroimmune signaling also regulates alcohol drinking behavior. Genetic comparisons among different strains of rats and mice found that addiction-like drinking behavior was associated with increased levels or activity of NF-κB, its regulatory proteins, and multiple innate immune genes (Mulligan et al. 2006). Furthermore, induction of innate immune genes resulted in increased ethanol consumption, whereas inactivation of such genes reduced drinking behavior (Blednov et al. 2005, 2011*b*). Thus, across genetically divergent strains of mice, innate immune responses to LPS corresponded to increases in ethanol consumption (Blednov et al. 2005, 2011*b*). In fact, even a single injection of LPS was able to produce a long-lasting increase in ethanol consumption (Blednov et al. 2011*a*) that corresponded to sustained increases in brain innate immune gene expression (Qin et al. 2007). These studies identified several innate immune molecules (e.g., β2-microglobulin, cathepsins, and CD14, a key innate immune signaling protein) as important for regulating drinking behavior. Thus, innate immune gene induction may underlie the progressive loss of behavioral flexibility, increasing negative affect, and increased alcohol drinking associated with repeat episodes of alcohol abuse and alcoholism.

## Activity of Innate Immune Genes Is Increased in the Addicted Brain

Direct analyses of changes in the activity or levels of various proteins in the brains of alcoholics and other drug addicts also can provide insight into the neurobiology of addiction. Such studies found the following:

- Postmortem studies of the brains of human alcoholics indicate that the innate immune chemokine MCP-1 is increased severalfold in multiple brain regions (Breese et al. 2008). Consistent with this, chronic alcohol treatment of mice (Qin et al. 2008) or of cultured brain slices from the rat hippocampus (Zou and Crews 2010) also increases expression of MCP-1 and other innate immune genes.
- Proteins that serve as markers of microglial activation are increased across the alcoholic brain (He and Crews 2008).
- Consistent with alcoholism being related to neuroimmune signaling, postmortem studies of gene expression in the brains of human alcoholics found increased levels of a subunit of NF-κB; moreover, 479 genes targeted by NF-κB showed increased expression in the frontal cortex of alcoholics (Okvist et al. 2007).
- Postmortem analyses of alcoholic human brain gene expression found innate immune activation of cell adhesion and extracellular membrane components of innate immune gene signaling (Liu et al. 2006).

Thus, the findings of several studies of gene or protein expression are consistent with increased neuroimmune signaling in the brains of addicted individuals.

## Polymorphisms of Innate Immune Genes and Genetic Risk of Addiction

Genetic factors account for approximately 50 percent of the risk of alcohol dependence (Schuckit 2009). Multiple genes linked to innate immune function also have been linked to the risk for alcoholism (see figure 3). DNA variations (i.e., polymorphisms) at specific locations on the chromosomes result in gene variants (i.e., alleles) that differ in their function or activity and thereby may increase or reduce the risk of alcoholism. For example, polymorphisms in the gene encoding an enzyme called CYP2E1, which is involved in ethanol metabolism, have been associated with the risk for alcoholism (Webb et al. 2010). In the body, CYP2E1 is highly expressed in monocyte-like cells; ethanol metabolism by CYP2E1 leads to the activation of these cells. Specifically, CYP2E1-mediated ethanol metabolism causes an increased production of highly reactive molecules called reactive oxygen species (ROS) within the monocytes that activate proinflammatory NF-κB responses (Cao et al. 2005) (see figure 3). In the brain, ethanol exposure leads to increased CYP2E1 expression, particularly in astrocytes (Montoliu et al. 1994, 1995), which likely contributes to astrocyte activation of NF-κB transcription during chronic alcohol exposure.

Human genetic association studies also have directly linked certain polymorphisms of the genes encoding NF-κB to alcohol dependence (Edenberg et al. 2008; Flatscher-Bader et al. 2005; Okvist et al. 2007). For example, polymorphisms in a precursor gene called *NF-kB1* that encodes one of the subunits of the transcription factor (i.e., the NF-κB p50 subunit) and which is important for activation of transcription have been associated with the risk for alcoholism (Edenberg et al. 2008). Likewise, alleles of the proinflammatory cytokine TNFα that result in increased TNFα expression have been linked to alcoholism and alcoholic liver disease (Pastor et al. 2000, 2005; Powell et al. 2000). Another genetic linkage exists between certain alleles of the anti-inflammatory, NF-κB–inhibiting cytokine IL-10 and alcoholism (Marcos et al.2008). Additional genetic evidence regarding innate immune genes and the risk for alcoholism comes from polymorphisms of the gene encoding a molecule called the IL-1 receptor antagonist as well as from multiple other alleles of the IL-1 gene complex (Saiz et al. 2009).

In general, gene polymorphisms associated with increased risk of alcoholism tend to increase proinflammatory responses. For example, alcohol exposure may increase the expression of proinflammatory cytokines or individuals at risk of alcohol dependence may carry alleles associated with decreased anti-inflammatory cytokine secretion. Thus, multiple innate immune gene polymorphisms are associated with genetic risk for alcoholism in humans, consistent with the assumption that increased brain innate immune gene expression contributes to the neurobiology of alcohol addiction.

## Summary

The findings summarized in this article link innate immune gene induction to addiction and alcoholism. Monocytes, microglia, and astrocytes are sensitive to AODs and stress, with repeated AOD use causing progressive innate immune gene induction that parallels changes in decision making, mood, and alcohol consumption. Stress and AODs activate NF-κB transcription in the brain, which in turn enhances expression of proinflammatory NF-κB target genes. As a result, molecules related to the innate immune response, such as the chemokine MCP-1, the proinflammatory cytokines TNFα, IL-1β, and IL-6; the proinflammatory oxidases iNOS, COX, and NOX (Qin et al. 2008); and proinflammatory proteases are found following chronic ethanol treatment. Postmortem analyses of human alcoholic brain also have demonstrated increased expression of innate immune genes, which can disrupt cognition, mood, and drug consumption and is consistent with addition-like behavior. Finally, polymorphisms of genes involved in the innate immune responses influence the risk for alcoholism. These studies suggest that innate immune genes contribute to alcoholism and may be involved in the genetic risk for alcoholism.

## Footnotes

1 Among the main molecules involved in the HPA system are the glucocorticoids (e.g., cortisol), and cycles of stress as well as AOD abuse lead to elevated basal glucocorticoid levels and promote addiction (Armario 2010).

2 Glutamate transporters are proteins that shuttle glutamate released by nerve cells (i.e., neurons) into the space between cells back into the neuron; this is essential to terminate transmission of a nerve signal and thus ensure appropriate regulation of neuronal activity.

3 Transcription factors are proteins that are necessary for a set of reactions called transcription, which is the first step of the process during which the genetic information encoded in the DNA is used as a template for the generation of functional proteins.

4 Prostaglandins are lipid compounds that are produced by almost all cells in the body and have a variety of important physiological effects, including the regulation of inflammatory reactions.

## References

[b1-arcr-34-3-355] Armario A (2010). Activation of the hypothalamic-pituitary-adrenal axis by addictive drugs: Different pathways, common outcome. Trends in Pharmacological Sciences.

[b2-arcr-34-3-355] Beattie MS, Ferguson AR, Bresnahan JC (2010). AMPA-receptor trafficking injury-induced cell death. European Journal of Neuroscience.

[b3-arcr-34-3-355] Bechara A, Dolan S, Hindes A (2002). Decision-making and addiction (part II): Myopia for the future or hypersensitivity to reward?. Neuropsychologia.

[b4-arcr-34-3-355] Blednov YA, Benavidez JM, Gell C (2011a). Activation of inflammatory signaling by lipopolysaccharide produces a prolonged increase of voluntary alcohol intake in mice. Brain, Behavior, and Immunity.

[b5-arcr-34-3-355] Blednov YA, Bergeson SE, Walker D (2005). Perturbation of chemokine networks by gene deletion alters the reinforcing actions of ethanol. Behavioural Brain Research.

[b6-arcr-34-3-355] Blednov YA, Ponomarev I, Geil C (2011b). Neuroimmune regulation of alcohol consumption: Behavioral validation of genes obtained from genomic studies. Addiction Biology.

[b7-arcr-34-3-355] Breese GR, Knapp DJ, Overstreet DH (2008). Repeated lipopolysaccharide (LPS) or cytokine treatments sensitize ethanol withdrawal-induced anxiety-like behavior. Neuropsychopharmacology.

[b8-arcr-34-3-355] Calu DJ, Roesch MR, Stalnaker TA, Schoenbaum G (2007). Associative encoding in posterior piriform cortex during odor discrimination and reversal learning. Cerebral Cortex.

[b9-arcr-34-3-355] Cao Q, Mak KM, Lieber CS (2005). Cytochrome P4502E1 primes macrophages to increase TNF-alpha production in response to lipopolysaccharide. American Journal of Physiology Gastrointestinal and Liver Physiology.

[b10-arcr-34-3-355] Chang EF, Merzenich MM (2003). Environmental noise retards auditory cortical development. Science.

[b11-arcr-34-3-355] Coleman LG, He J, Lee J (2011). Adolescent binge drinking alters adult brain neurotransmitter gene expression, behavior, brain regional volumes, and neurochemistry in mice. Alcoholism: Clinical and Experimental Research.

[b12-arcr-34-3-355] Crews FT, Bechara R, Brown LA (2006). Cytokines and alcohol. Alcoholism: Clinical and Experimental Research.

[b13-arcr-34-3-355] Crews F, Nixon K, Kim D (2006). BHT blocks NF-kappaB activation and ethanol-induced brain damage. Alcoholism: Clinical and Experimental Research.

[b14-arcr-34-3-355] Crews FT, Zou J, Qin L (2011). Induction of innate immune genes in brain create the neurobiology of addiction. Brain, Behavior, and Immunity.

[b15-arcr-34-3-355] Edenberg HJ, Xuei X, Wetherill LF (2008). Association of NFKB1, which encodes a subunit of the transcription factor NF-kappaB, with alcohol dependence. Human Molecular Genetics.

[b16-arcr-34-3-355] Eisenberger NI, Berkman ET, Inagaki TK (2010). Inflammation-induced anhedonia: Endotoxin reduces ventral striatum responses to reward. Biological Psychiatry.

[b17-arcr-34-3-355] Flatscher-Bader T, van der Brug M, Hwang JW (2005). Alcohol-responsive genes in the frontal cortex and nucleus accumbens of human alcoholics. Journal of Neurochemistry.

[b18-arcr-34-3-355] Ghosh S, Hayden MS (2008). New regulators of NF-kappaB in inflammation. Nature Reviews Immunology.

[b19-arcr-34-3-355] Graeber MB (2010). Changing face of microglia. Science.

[b20-arcr-34-3-355] He J, Crews FT (2008). Increased MCP-1 and microglia in various regions of the human alcoholic brain. Experimental Neurology.

[b21-arcr-34-3-355] Kelley KW, Dantzer R (2011). Alcoholism and inflammation: Neuroimmunology of behavioral and mood disorders. Brain, Behavior, and Immunity.

[b22-arcr-34-3-355] Knapp DJ, Crews FT (1999). Induction of cyclooxygenase-2 in brain during acute and chronic ethanol treatment and ethanol withdrawal. Alcoholism: Clinical and Experimental Research.

[b23-arcr-34-3-355] Koo JW, Russo SJ, Ferguson D (2010). Nuclear factor-kappaB is a critical mediator of stress-impaired neurogenesis and depressive behavior. Proceedings of the National Academy of Sciences of the United States of America.

[b24-arcr-34-3-355] Liu J, Lewohl JM, Harris RA (2006). Patterns of gene expression in the frontal cortex discriminate alcoholic from nonalcoholic individuals. Neuropsychopharmacology.

[b25-arcr-34-3-355] Madrigal JL, Garcia-Bueno B, Moro MA (2003). Relationship between cyclooxygenase-2 and nitric oxide synthase-2 in rat cortex after stress. European Journal of Neuroscience.

[b26-arcr-34-3-355] Madrigal JL, Moro MA, Lizasoain I (2002). Stress-induced increase in extracellular sucrose space in rats is mediated by nitric oxide. Brain Research.

[b27-arcr-34-3-355] Marcos M, Pastor I, Gonzalez-Sarmiento R, Laso FJ (2008). Interleukin-10 gene polymorphism is associated with alcoholism but not with liver disease. Alcohol and Alcoholism.

[b28-arcr-34-3-355] Montoliu C, Sancho-Tello M, Azorin I (1995). Ethanol increases cytochrome P4502E1 and induces oxidative stress in astrocytes. Journal of Neurochemistry.

[b29-arcr-34-3-355] Montoliu C, Valles S, Renau-Piqueras J, Guerri C (1994). Ethanol-induced oxygen radical formation and lipid peroxidation in rat brain: Effect of chronic alcohol consumption. Journal of Neurochemistry.

[b30-arcr-34-3-355] Mulligan MK, Ponomarev I, Hitzemann RJ (2006). Toward understanding the genetics of alcohol drinking through transcriptome meta analysis. Proceedings of the National Academy of Sciences of the United States of America.

[b31-arcr-34-3-355] Munhoz CD, Sorrells SF, Caso JR (2010). Glucocorticoids exacerbate lipopolysac-charide-induced signaling in the frontal cortex and hippocampus in a dose-dependent manner. Journal of Neuroscience.

[b32-arcr-34-3-355] Obernier JA, White AM, Swartzwelder HS, Crews FT (2002). Cognitive deficits and CNS damage after a 4-day binge ethanol exposure in rats. Pharmacology, Biochemistry, and Behavior.

[b33-arcr-34-3-355] Okvist A, Johansson S, Kuzmin A (2007). Neuroadaptations in human chronic alcoholics: Dysregulation of the NF-kappaB system. PLoS One.

[b34-arcr-34-3-355] Pascual M, Balino P, Alfonso-Loeches S (2011). Impact of TLR4 on behavioral and cognitive dysfunctions associated with alcohol-induced neuroinflammatory damage. Brain, Behavior, and Immunity.

[b35-arcr-34-3-355] Pascual M, Blanco AM, Cauli O (2007). Intermittent ethanol exposure induces inflam-matory brain damage and causes long-term behavioural alterations in adolescent rats. European Journal of Neuroscience.

[b36-arcr-34-3-355] Pastor IJ, Laso FJ, Avila JJ (2000). Polymorphism in the interleukin-1 receptor antagonist gene is associated with alcoholism in Spanish men. Alcoholism: Clinical and Experimental Research.

[b37-arcr-34-3-355] Pastor IJ, Laso FJ, Romero A, Gonzalez-Sarmiento R (2005). -238 G>A polymorphism of tumor necrosis factor alpha gene (TNFA) is associated with alcoholic liver cirrhosis in alcoholic Spanish men. Alcoholism: Clinical and Experimental Research.

[b38-arcr-34-3-355] Powell EE, Edwards-Smith CJ, Hay JL (2000). Host genetic factors influence disease progression in chronic hepatitis C. Hepatology.

[b39-arcr-34-3-355] Qin L, He J, Hanes RN (2008). Increased systemic and brain cytokine production and neuroinflammation by endotoxin following ethanol treatment. Journal of Neuroinflammation.

[b40-arcr-34-3-355] Qin L, Wu X, Block ML (2007). Systemic LPS causes chronic neuroinflammation and progressive neurodegeneration. Glia.

[b41-arcr-34-3-355] Saiz PA, Garcia-Portilla MP, Florez G (2009). Polymorphisms of the IL-1 gene complex are associated with alcohol dependence in Spanish Caucasians: Data from an association study. Alcoholism: Clinical and Experimental Research.

[b42-arcr-34-3-355] Schoenbaum G, Shaham Y (2008). The role of orbitofrontal cortex in drug addiction: A review of preclinical studies. Biological Psychiatry.

[b43-arcr-34-3-355] Schoenbaum G, Roesch MR, Stalnaker TA (2006). Orbitofrontal cortex, decision-making and drug addiction. Trends in Neurosciences.

[b44-arcr-34-3-355] Schoenbaum G, Saddoris MP, Ramus SJ (2004). Cocaine-experienced rats exhibit learning deficits in a task sensitive to orbitofrontal cortex lesions. European Journal of Neuroscience.

[b45-arcr-34-3-355] Schuckit MA (2009). An overview of genetic influences in alcoholism. Journal of Substance Abuse Treatment.

[b46-arcr-34-3-355] Stalnaker TA, Takahashi Y, Roesch MR, Schoenbaum G (2009). Neural substrates of cognitive inflexibility after chronic cocaine exposure. Neuropharmacology.

[b47-arcr-34-3-355] Webb A, Lind PA, Kalmijn J (2011). The investigation into CYP2E1 in relation to the level of response to alcohol through a combination of linkage and association analysis. Alcoholism: Clinical and Experimental Research.

[b48-arcr-34-3-355] Zou JY, Crews FT (2005). TNF alpha potentiates glutamate neurotoxicity by inhibiting glutamate uptake in organotypic brain slice cultures: Neuroprotection by NF kappa B inhibition. Brain Research.

[b49-arcr-34-3-355] Zou J, Crews F (2006). CREB and NF-kappaB transcription factors regulate sensitivity to excitotoxic and oxidative stress induced neuronal cell death. Cellular and Molecular Neurobiology.

[b50-arcr-34-3-355] Zou J, Crews F (2010). Induction of innate immune gene expression cascades in brain slice cultures by ethanol: Key role of NF-kappaB and proinflammatory cytokines. Alcoholism: Clinical and Experimental Research.

